# Sodium Imbalance in Mice Results Primarily in Compensatory Gene Regulatory Responses in Kidney and Colon, but Not in Taste Tissue

**DOI:** 10.3390/nu12040995

**Published:** 2020-04-03

**Authors:** Kristina Lossow, Wolfgang Meyerhof, Maik Behrens

**Affiliations:** 1 Molecular Genetics, German Institute of Human Nutrition Potsdam-Rehbruecke, Arthur-Scheunert-Allee 114-116, 14558 Nuthetal, Germany; kristina.lossow@uni-jena.de (K.L.); wolfgang.meyerhof@uks.eu (W.M.); 2 Department of Molecular Nutritional Physiology, Institute of Nutritional Sciences, Friedrich Schiller University Jena, Dornburger Str. 24, 07743 Jena, Germany; 3 Center for Integrative Physiology and Molecular Medicine, Saarland University, Kirrbergerstr., Bldg. 48, 66421 Homburg, Germany; 4 Leibniz-Institute for Food Systems Biology at the Technical University of Munich, Lise-Meitner-Str. 34, 85354 Freising, Germany

**Keywords:** epithelial sodium channel, sodium homeostasis, amiloride, salt deprivation, short-term preference test

## Abstract

Renal excretion and sodium appetite provide the basis for sodium homeostasis. In both the kidney and tongue, the epithelial sodium channel (ENaC) is involved in sodium uptake and sensing. The diuretic drug amiloride is known to block ENaC, producing a mild natriuresis. However, amiloride is further reported to induce salt appetite in rodents after prolonged exposure as well as bitter taste impressions in humans. To examine how dietary sodium content and amiloride impact on sodium appetite, mice were subjected to dietary salt and amiloride intervention and subsequently analyzed for ENaC expression and taste reactivity. We observed substantial changes of ENaC expression in the colon and kidney confirming the role of these tissues for sodium homeostasis, whereas effects on lingual ENaC expression and taste preferences were negligible. In comparison, prolonged exposure to amiloride-containing drinking water affected β- and αENaC expression in fungiform and posterior taste papillae, respectively, next to changes in salt taste. However, amiloride did not only change salt taste sensation but also perception of sucrose, glutamate, and citric acid, which might be explained by the fact that amiloride itself activates bitter taste receptors in mice. Accordingly, exposure to amiloride generally affects taste impression and should be evaluated with care.

## 1. Introduction

Sodium is the main cation in the extracellular fluid and the primary determinant of osmolarity, crucial for many biological processes [1]. Accordingly, levels of sodium are tightly controlled through a precise balance of sodium intake and excretion. The latter is primarily realized via the kidney, which plays a major role in volume, electrolyte, and blood pressure regulation [2,3]. To move sodium across the apical plasma membrane, the distal tubules and collecting duct of the kidney utilize the epithelial sodium channel (ENaC). In times of sodium deficits, aldosterone, a mineralocorticoid hormone produced in the adrenal glands [4], promotes the translocation of ENaC from cytoplasmic compartments to the apical plasma membrane in the renal collecting system [5] triggering sodium reabsorption. In comparison, sodium absorption in the intestine is primarily realized by the sodium/hydrogen exchanger rather than through the ENaC, which is limited to epithelial cells of the distal colon and rectum [6,7]. However, after proctocolectomy, ENaC was verified in the distal part of the small intestine, indicating the importance of ENaC-mediated reabsorption of salt and water in the intestine [8]. Inhibition of ENaC is achieved by amiloride [9,10,11,12], first described by Cragoe in 1967 [13]. Amiloride lowers systemic blood pressure by preventing absorption of sodium and increasing its excretion along with water (natriuresis) [14,15,16].

Additionally, sodium homeostasis is maintained by ingestive behavior. Sodium appetite is the instinctive drive to seek salty substances or beverages for consumption [17] stimulated by sodium deficiency, hypovolemia, or mineralocorticoids. Accordingly, sodium-depleted rats ingest high sodium chloride solutions even at concentrations they would normally reject [18,19,20,21]. Already decades ago, studies revealed that amiloride not only affects the kidney but also acts as a potent blocker of salt taste in rodents [22,23,24,25]. Applied to the tongue before or during sodium stimulation, amiloride reduces sodium-evoked responses in the chorda tympani nerve innervating the fungiform papillae on the frontal tongue as well as the palate in the oral cavity [9,10,26,27]; vice versa, sodium deprivation or aldosterone application results in an increased number of amiloride-sensitive taste receptor cells in fungiform papillae [28].

Conclusively, ENaC has been detected in lingual epithelia and taste buds [9,10,23,29,30,31,32,33]. Fully functional ENaC is believed to be composed of three homologous subunits (α, β, and γ) arranged with a 1:1:1 stoichiometry [34,35]. In 2010, Chandrashekar and colleagues demonstrated the involvement of the α-subunit of ENaC in salt attraction and sodium taste responses by a tissue-specific knock-out model [32]. However, a recent gene-targeted approach, using fluorescent marker proteins under the control of the *Scnn1a* and *Scnn1b* gene loci, encoding α- and βENaC, respectively, concluded that the assumed αβγ-subunit composition of ENaC seems highly unlikely in taste tissue, as ENaC subunits were distributed in different taste bud cells under adequate sodium conditions [33].

Accordingly, the cellular and molecular composition of the “salt taste receptor” is still quite controversial with many unanswered questions. Furthermore, studies regarding the expression levels of ENaC subunits in gustatory tissues after dietary sodium restriction are rare and inconclusive [28,36]. To explore how sodium ingestion as well as short- and longtime exposure to amiloride impact on sodium appetite, we utilized a gene-targeted animal model with modified *Scnn1a* and *Scnn1b* loci, which were subjected to dietary and amiloride interventions, followed by taste reactivity testing (short-term preference test) and expression analysis of potential ENaC subunits.

## 2. Materials and Methods

### 2.1. Animal Experiments

All animal experiments were approved by and conducted following the national guidelines of the Ministry of Environment, Health and Consumer Protection of the federal state of Brandenburg (14513 Teltow, Germany; 2347-02-2017), and institutional guidelines of the German Institute of Human Nutrition Potsdam-Rehbruecke (14558 Nuthetal, Germany; T-01-17, T-02-17). Mice were housed in polycarbonate cages and kept under constant conditions (12 h light/dark cycles with light beginning at 6:00 am; 22 °C room temperature; 55% humidity). The animals received food and water *ad libitum* (for details see feeding experiments).

### 2.2. Feeding Experiment and Amiloride Intervention

In this study, either wild-type or homozygous double gene-targeted Scnn1a*^IRES-GFP^*/Scnn1b*^IRES-tdRFP^* mice (abbreviated Scnn1^++/++^ and Scnn1^aa/bb^, respectively, throughout) with modified *Scnn1a* and *Scnn1b* loci were used. These carried modified alleles allowing the synthesis of green (GFP) and red (tdRFP) fluorescent proteins in cells expressing α- and βENaC subunits, respectively. Details of modification/gene-targeting were described previously [33].

Before the experiments, Scnn1^++/++^ and Scnn1^aa/bb^ animals had free access to water and standard chow diet (V1534, Ssniff, Soest, Germany; sodium content of 0.24%). From the 7th week of life on, the animals continued to have free access to water, while the chow was replaced either by a sodium-adequate (equal sodium content as within the chow, however 4.8 above nutrient requirements of laboratory animals) [37], sodium-deficient or high salt diet, with 0.21% (0.5% NaCl; E15430-04, Ssniff, Soest, Germany), <0.03% (E15430-24, Ssniff, Soest, Germany), and 1.71% sodium (4% NaCl; E15431-34, Ssniff, Soest, Germany), respectively. Body weight, food, and water intake were monitored weekly from weaning onward (21 days). Blood pressure was recorded prior to diet change/experimental session (6th week) and after 4 weeks of intervention (10th week) using Power Lab and Chart 5 software (ADInstruments Ltd., Oxford, UK). Urine samples were collected during the day (6 am to 5 pm) and overnight (5 pm to 6 am) at week 10, while mice were individually housed in metabolic cages (TechniPlast, Buguggiate, Italy). Urinary sodium content was determined in triplicate with a LAQUA twin system (Horiba Scientific for Na+, Darmstadt, Germany). Until then, all animals went through the same experimental procedure differing only in the sodium content of the diet. To test the effect of the consumed sodium on RNA expression levels and the distribution of fluorescent proteins, a randomly selected experimental set of mice were killed at the 11th week of life and subjected either to taste bud isolation (*n* = 4–6 per diet and genotype) or perfusion (*n* = 3 per diet and genotype). In a different experimental set, taste responses to different taste solutions were assessed by a short-term preference test. The remaining mice further continued with the dietary intervention for additional 4 weeks (*n* = 10–11 per diet and genotype) with defined access to water and food (for details see short-term preference test). In the third experimental setup, taste responses to different taste solutions after pre-treatment with amiloride were examined. In this experiment, mice fed a sodium-adequate diet either received access to amiloride-containing (300 µM) or amiloride-free water for 36 h [38] prior to short-term preference tests (*n* = 10–16 per intervention and genotype). Amiloride treatment was restricted to a maximum of 3 times for each animal, with a recovery phase of 36 h in-between (with amiloride-free water). After the final intervention/third amiloride treatment, animals were subjected either to taste bud isolation (*n* = 4 per intervention and genotype) or perfusion (*n* = 3 per intervention and genotype).

### 2.3. Short-term Preference Tests

Short-term preference tests were performed using the DavisRig system (MS-160, DiLog Instruments, Tallahassee, FL, USA), permitting several taste stimuli to be presented in a brief trial within a single test session. During these experiments, mice were singly housed with defined access to water and food. Mice were initially trained for 3 days after 18 h water restriction, using water as test stimulus to get used to the shutter system. To monitor attractive taste responses, animals were restricted for 22.5 h with limited access to food (1 g of the corresponding diet) and water (2.5 mL). For aversive taste stimuli, mice were water restricted over a period of 22.5 h with free access to food, as reported earlier [39]. Mice performed preference tests at the beginning of the light phase, followed by 1 h with free access to water and food. Test sessions were restricted to 2 or 5 consecutive days for attractive or aversive taste stimuli, respectively. To control for small individual differences in lick rate, number of licks to each taste stimulus was divided by the average number of licks to water alone for each mouse and day. A lick ratio of 1.0 reveals that licks to taste stimulus equals licks to water. In contrast, a lick ratio close to 0 indicates only a few licks of taste stimulus relative to licks for water, and a lick ratio > 1.0 signifies more licks of the taste stimulus relative to that for water. In case of apparent decreasing motivation of the animals, for example, reduced water licks under aversive test conditions or diminished licks for sucrose and sucralose under attractive restriction conditions, we excluded the entire subsequent test session. Accordingly, sometimes only the first 15 instead of 20 min were taken into consideration, adjusted day by day, and according to the motivational behavior of each mouse.

Taste solutions for testing were prepared daily with reagent grade chemicals and distilled water, sucrose (10–1000 mM, Merck, Darmstadt, Germany), monopotassium glutamate (MPG; 1–100 mM; Fluka, Oberhaching, Germany), denatonium benzoate (DB; 0.1–10 mM; Sigma, Taufkirchen, Germany), citric acid (CA; 1–100 mM; Roth, Karlsruhe, Germany), sodium chloride (NaCl; 10–1000 mM; Roth, Karlsruhe, Germany), inosine monophosphate (IMP; 1 mM; Sigma, Taufkirchen, Germany), and amiloride (0.1 mM; Sigma, Taufkirchen, Germany). Solutions were presented at room temperature. The sequence of stimulus presentation was randomized with varying taste solutions and concentrations (no ascending or descending presentation of a single taste solution) every day to minimize systematic order and contrast effects.

### 2.4. Tissue Preparation

Scnn1^++/++^ and Scnn1^aa/bb^ mice were either anesthetized by intraperitoneal injection of 150 mg/kg body weight pentobarbital (Narcoren from Merial, Hallbergmoos, Germany) followed by transcardial perfusion with phosphate-buffered saline (PBS; 0.01 M Na_2_HPO_4_, 1.764 mM KH_2_PO_4_, 2.683 mM KCl, 0.1369 M NaCl, pH 7.4) and 4% paraformaldehyde (PFA) to gain tissue for cryosections (14 µm) and subsequent immunohistochemistry or with isoflurane (Cp-pharma, Burgsdorf, Germany) to gain tissues for RNA extractions as reported earlier [33].

### 2.5. Immunohistochemistry

Immunohistochemistry was performed as described before [33]. Primary antibodies included TrpM5 (1:5000; [40]) and aromatic L-amino acid decarboxylase (AADC, also known as DOPA decarboxylase, 1:500; GTX30448, GeneTex, Irvine, CA, USA).

Co-localization analysis in fungiform papillae was based on confocal (Leica TCS SP8) z-stack scans through tissue sections, of which a single plane was used for evaluation. Mean fluorescence intensity was evaluated in the operating mode of TCS SP8 LAS X software by defining a region of interest (ROI), namely one taste bud, in the digitized graph. Collected data reflect results from tissue sections of at least 3 mice per intervention.

### 2.6. RNA Isolation and qRT-PCR

Taste cells, lingual epithelium, kidney, and distal colon were subjected to RNA isolation and cDNA synthesis as previously reported [33]. Afterwards, 12.5 ng/well cDNA served as a template for quantitative RT-PCR using TaqMan Gene Expression Master Mix (Applied Biosystems by ThermoFisher Scientific, Foster City, CA, USA) and the corresponding probes (αENaC, Mm00803386_m1; βENaC, Mm00441215_m1; γENaC, Mm00441228_m1; Eef2, Mm01171435_gH; all ABI Applied Biosystems by ThermoFisher Scientific, Foster City, CA, USA; β-actin probe 5′FAM-TTGAGACCTTCAACACCCCAGCCA-3′TAM, β-actin for 5′-TACGACCAGAGGCATACAG-3′, β-actin rev 5′-GCCAACCGTGAAAAGATGAC-3′; Eurofins MWG Operon, Martinsried, Germany) in a final volume of 10 µL. After initial 10 min denaturation at 95 °C, PCR was carried out for 40 cycles with 95 °C for 15 s and 60 °C for 60 s using Quant Studio 12K Flex Real-Time PCR System (Applied Biosystems by ThermoFisher Scientific, Foster City, CA, USA). Relative expression was determined based on normalization to β-actin and eukaryotic translation elongation factor 2 (Eef2) mRNA levels. As amplification efficiencies of all used probes were quite similar, cycle threshold (CT) values were averaged from triplicates and differences between CT values of ENaC and the housekeeping genes were calculated as ΔCT for normalization and finally expressed as 2^−ΔCT^. Collected data reflect results from isolated tissue of at least 4 mice.

### 2.7. Functional Expression Analysis

Functional expression analysis of bitter taste receptor cDNAs has been reported in detail earlier [39]. For the screening for amiloride-responsive bitter taste receptors, the compound has been applied to human embryonal kidney cells transiently expressing mouse (Tas2rs) and human bitter taste receptors (TAS2Rs) at a concentration of 3 mM. Subsequently, hits were verified by dose-concentration analyses, with concentrations ranging between 0.0001 and 3 mM. Latter data reflect results from 3 individual experiments.

### 2.8. Statistics

Data are given as means ± standard deviation (STABW; tables) or standard error (STE; figures). Statistical reliability of the observed results in the feeding study was determined by UNIANOVA and post-hoc analysis using Bonferroni’s multiple comparison test (SPSS, SPSS 16.0, IBM, Chigago, IL, USA). For quantitative expression analysis, significant differences between 3 diets were determined by UNIANOVA (SPSS, SPSS 16.0, IBM, Chicago, IL, USA), followed by a comparison of individual pairs of means using Bonferroni’s post-hoc test, whereas comparison between 2 intervention groups was conducted by using Student’s unpaired *t*-test. Differences were considered to be statistically significant if *p* ≤ 0.05.

## 3. Results

### 3.1. Feeding Experiment

**Figure 1:**
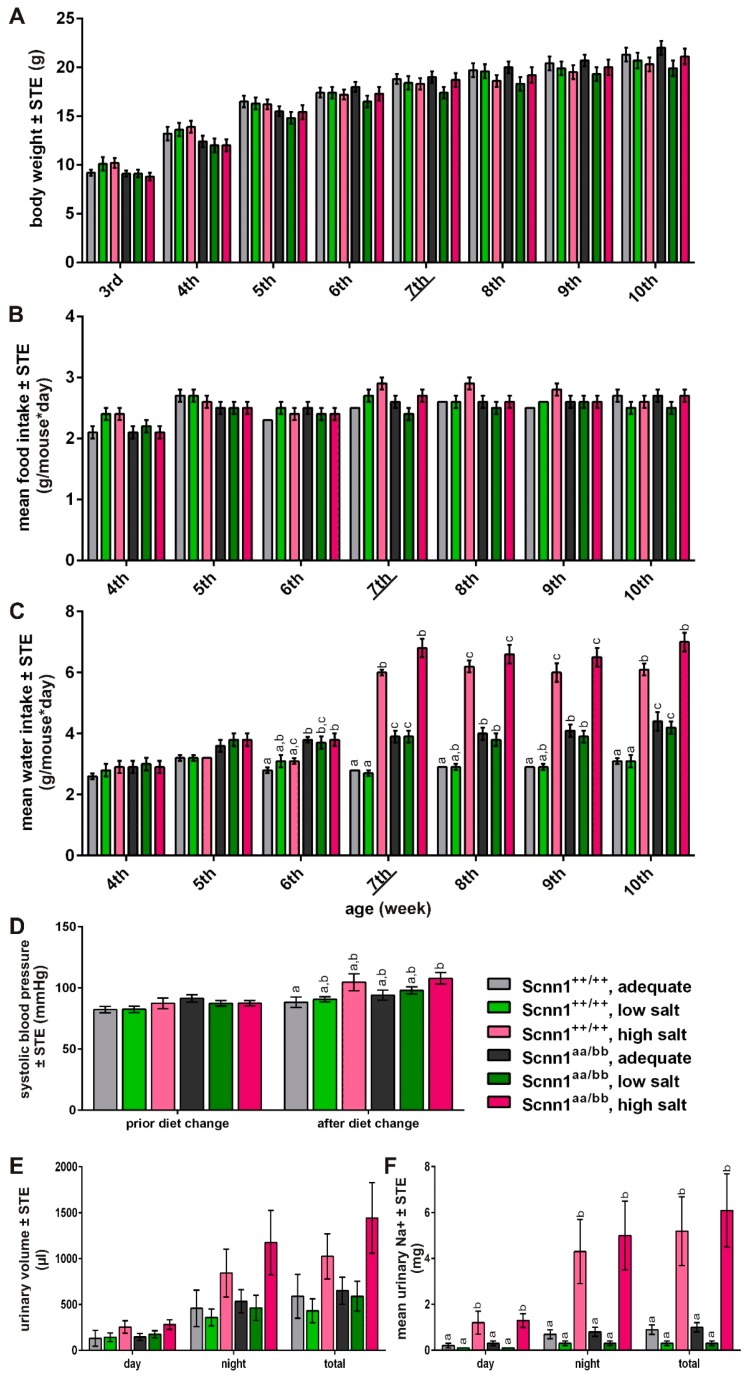
Physiological parameters of Scnn1^++/++^ and Scnn1^aa/bb^ mice during dietary monitoring. (**A**) Body weight, (**B**) food and (**C**) water intake, (**D**) blood pressure, (**E**) urinary volume, and (**F**) sodium excretion of Scnn1^++/++^ (*n* = 11) and Scnn1^aa/bb^ mice (*n* = 19–20) fed with low salt, adequate, and high salt diet over a period of 4 weeks (7th to 10th week, time point of diet change is indicated by underline); after initial 3 weeks of sodium adequate chow diet (4th to 6th week) after weening (3rd week). Statistical differences between 2 bars at a specific time point are indicated by different letters based on UNIANOVA and post-hoc analysis using Bonferroni´s multiple comparison test.

To investigate if diets with varying sodium content impact on the expression of ENaC, we fed Scnn1^++/++^ and Scnn1^aa/bb^ mice with low, adequate, and high salt diets over a period of 4 weeks (from 7th to 10th week of age). We monitored body weight, food, and water intake from weaning onward. As expected, body weight constantly increased during the experiment independent of diet and genotype (*p* < 0.001; Figure 1A). Mice consumed equal amounts of food, ranging from 1.3 g after weaning to 3.7 g, with a mean intake of 2.5 g per day, irrespective of the sodium content of the diet (Figure 1B). In contrast, water intake changed after the diet switched (Figure 1C). When fed a high salt diet, mice increased their daily water intake by about 78% in comparison to animals fed with a diet adequate in sodium. The water intake of mice on low, adequate, and high salt diet ranged around 3.6 ± 0.5 mL, 3.6 ± 0.6 mL, and 6.5 ± 0.7 mL per day, respectively (*p* < 0.001). Furthermore, we observed significant differences in water intake based on genotype from the 5th week on, with significantly higher values in Scnn1^aa/bb^ animals (Figure 1C).

In addition, we measured blood pressure prior and at the end of the dietary intervention (Figure 1D). Prior to diet change, no significant differences for mean systolic blood pressure were observed. After 3.5 weeks, animals that received the high salt diet showed slightly increased systolic blood pressure compared to animals that ate low or adequate salt diets, triggering significant diet X genotype effects (*p* = 0.012). Furthermore, urine was collected in metabolic cages at the end of the dietary intervention. Urinary volumes of mice fed the high salt diet were slightly higher, relative to the other groups. However, differences did not reach significance when considered for day, night, or total volume, due to high variations between the animals (Figure 1E). Considering mean urinary sodium content, significant variances were observed depending on diet (significant differences between all groups) and diet X genotype (*p* < 0.001), but not based on genotype itself. Thereby, no significant differences between groups fed low and sodium-adequate diet were recognized, whereas animals receiving the high salt diet showed significantly higher urinary sodium content in comparison to all other groups (Figure 1F).

### 3.2. Expression Analysis

In order to examine the impact of dietary salt on the expression of the ENaC subunits, we measured the transcription levels in the gustatory tissue, kidney, and distal colon of Scnn1^aa/bb^ mice. Quantitative RT-PCR analyses of isolated fungiform and foliate/vallate taste buds and of non-gustatory lingual tissue showed no differences in relative expression levels of all 3 ENaC subunits after feeding a high or low salt diet in comparison to Scnn1^aa/bb^ animals fed a control diet over a period of 4 weeks (Table 1). In kidney, αENaC expression was significantly reduced after consumption of a high salt diet, whereas β- and γENaC were not affected (Table 1). In the distal colon, intervention with a low salt diet resulted in significantly higher expression levels of α- and βENaC in comparison to Scnn1^aa/bb^ animals fed with a standard or high salt diet. For γENaC, such significant variances were only detected between Scnn1^aa/bb^ animals fed a low and high salt diet, with higher expression levels for animals who received a low salt diet (Table 1).

**Figure 2:**
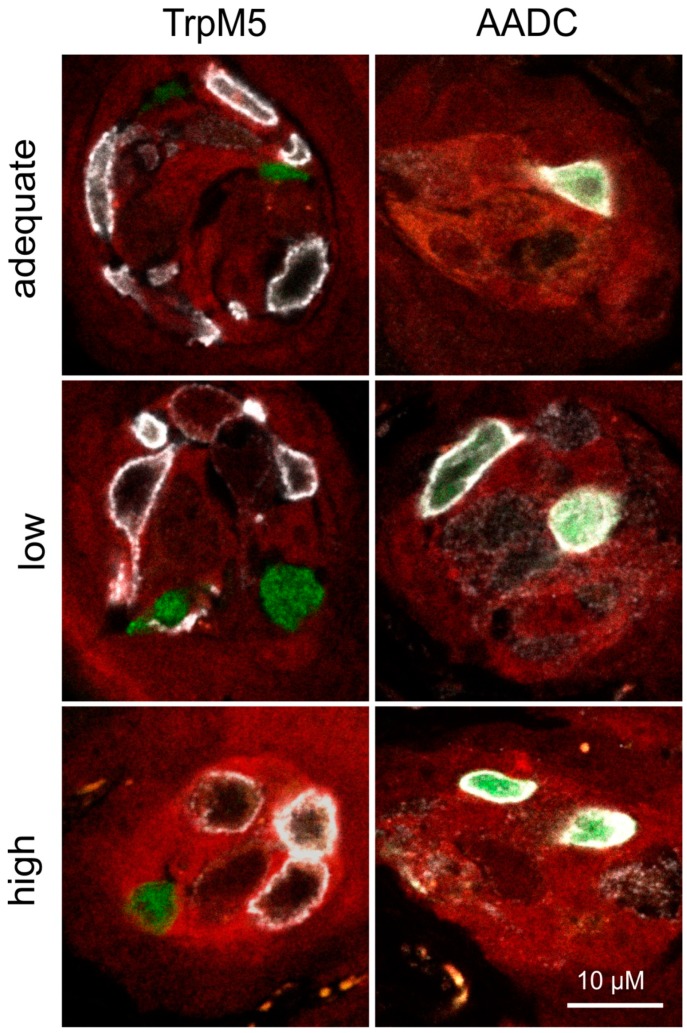
Expression of fluorescent proteins in fungiform papillae after dietary intervention. Fungiform papillae sections of Scnn1^aa/bb^ animals expressing GFP (synthesis of green) and tdRFP (synthesis of red) fluorescence in αENaC- and βENaC-expressing cells, respectively, were stained for Type II (TrpM5) and Type III (AADC) taste cell markers after dietary intervention. Therefore, animals received either an adequate, low, or high salt diet over a period of 4 weeks. Independently of the consumed diet, GFP and tdRFP fluorescence showed no co-localization in taste papillae. Whereas GFP-positive cells always co-expressed AADC, tdRFP-positive cells revealed no overlap with the cell markers TrpM5 or AADC, visualized by immunofluorescence (white). Scale bars apply to all images.

Furthermore, no differences were noted regarding expression of the fluorescent reporter proteins in fungiform taste buds of Scnn1^aa/bb^ animals. Consumption of either the low or high salt diet did not affect the location of GFP and tdRFP fluorescence, nor the fluorescence intensity. Accordingly, GFP always co-localized with Type III cell marker AADC, whereas tdRFP was not co-expressed with Type II or Type III cell markers TrpM5 or AADC, respectively (Figure 2).

However, ENaC expression of Scnn1^aa/bb^ in comparison to Scnn1^++/++^ animals consuming an adequate salt diet revealed overall higher expression levels (Table S1). Whereas non-gustatory tissue differences in the expression levels of ENaC subunits did not reach statistical significance, prominent differences were recognized in lingual taste papillae affecting all ENaC subunits. In kidney, genotype primarily affected α- and βENaC subunits, whereas in the distal colon only βENaC expression was significantly increased in Scnn1^aa/bb^ animals.

### 3.3. Short-term Preference Tests

**Figure 3:**
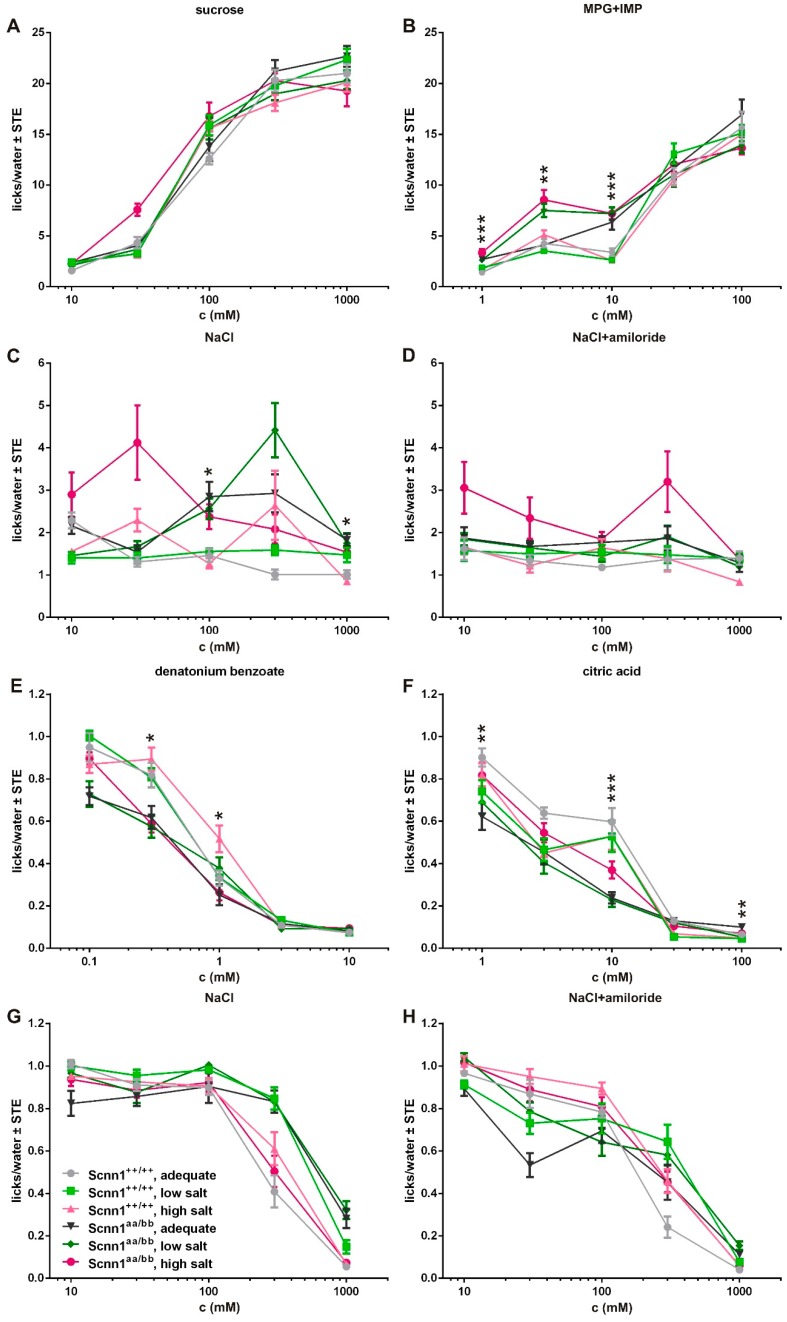
Taste response curves of Scnn1^++/++^ and Scnn1^aa/bb^ mice after dietary intervention. After 4 weeks fed with sodium-adequate, low, or high salt diet, Scnn1^++/++^ and Scnn1^aa/bb^ mice were subjected to short-term preference tests using an automated gustometer. To do so, animals were either restricted for 22.5 h with access to 2.0 mL water and 1 g food (attractive restriction conditions, (**A**–**D**)) or water-deprived for 22.5 h (aversive restriction conditions, (**E**–**H**)). Taste solutions and concentrations were presented randomly. Each data point represents a mean ± standard error (SE) of 5 s presentations from the 10 to 11 animals tested. Statistical testing was based on UNIANOVA and post-hoc analysis using Bonferroni´s multiple comparison test. Significant differences over all groups in line drawings were indicated by asterisk(s) with * *p* < 0.05, ** *p* < 0.01, and *** *p* < 0.001.

In order to determine if the sodium content of diets affects taste preferences for NaCl or other tastants, despite the lack of changes in gustatory ENaC expression, we performed a dietary intervention study. A group of 10 to 11 Scnn1^++/++^ and Scnn1^aa/bb^ mice was fed an adequate, low, or high salt diet for another 4 weeks (total intervention of 8 weeks, from week 7–15). During this time, short-term preference tests were performed under conditions allowing the analysis of taste responses to attractive (Figure 3A–D) and aversive (Figure 3E–H) taste stimuli. For this procedure, it was mandatory to deprive animals of water. Even though the mice that received the high salt diet drank significantly more water under *ad libitum* conditions (Figure 1C), water restriction did not have an impact on the water lick rates during 5 s measuring periods. Accordingly, taste preference tests were carried out under the same conditions for all animals, independent of diet or genotype. Mean lick responses are shown in Figure 3.

Under conditions for testing attractive stimuli there was no diet X genotype effect for sucrose (Table S3). However, ANOVA revealed an effect for concentration with increasing number of licks/water at 30 mM sucrose for Scnn1^aa/bb^ animals under the high salt regime (Figure 3A). For MPG+IMP, there were effects for concentration and diet X genotype (Figure 3B, Tables S2 and S3). The latter was observed at 1.0, 3.0, and 10 mM, with Scnn1^aa/bb^ animals showing increased lick rates (Table S2). NaCl taste solutions were licked more at the low concentration of 30 mM by Scnn1^aa/bb^ animals under the high salt regime and resulted in higher lick numbers at the high salt concentration of 300 mM by Scnn1^aa/bb^ animals pretreated according to the low salt regime (Figure 3C, Tables S2 and S3). These effects were eliminated by amiloride treatment (Figure 3D, Tables S2 and S3). Control stimuli like IMP and denatonium benzoate presented only at a single concentration did not result in any diet X genotype effects (Figure S1A, Tables S2 and S3). In comparison to that, the control stimuli amiloride and citric acid revealed such effects, with higher lick/water ratios in Scnn1^aa/bb^ animals (Figure S1A, Tables S2 and S3). For amiloride, highest lick/water means were observed for Scnn1^aa/bb^ animals fed with a high salt diet, which were even higher than for animals fed the low or adequate diet, indicating that in this case not only genotype but also diet has an effect.

Additionally, animals were further water-restricted for 22.5 h to perform short-term preference tests for aversive stimuli (Figure 3E–H, Tables S2 and S3). Here, all taste stimuli were affected by concentration and diet X genotype (Table S3). For denatonium benzoate, Scnn1^++/++^ mice showed higher mean licks for denatonium benzoate at 0.1 to 1.0 mM than other diet X genotype constellations (Figure 3E). Scnn1^++/++^ animals further tended to show higher lick to water ratios for citric acid at 1 and 10 mM (exception: low salt fed animals at 1 mM; Figure 3F). In comparison to that, NaCl did not result in significant differences at 10 to 100 mM, whereas Scnn1^aa/bb^ and Scnn1^++/++^ mice fed with a low salt diet and Scnn1^aa/bb^ animals fed with an adequate diet showed higher lick ratios than Scnn1^++/++^ animals fed with an adequate diet or animals fed with a high salt diet (both genotypes; Figure 3G). Independent of diet, we observed changes in lick ratios among Scnn1^++/++^ and Scnn1^aa/bb^ animals for 1 mM MPG+IMP, 100–1000 mM NaCl (attractive regime) as well as for 10 mM and 100 mM citric acid, 300–1000 mM NaCl, and 1000 mM NaCl+amiloride (aversive regime), respectively, when testing diet adequate in sodium content (*p* ≤ 0.05, Student’s *t*-test).

### 3.4. Amiloride Intervention

**Figure 4:**
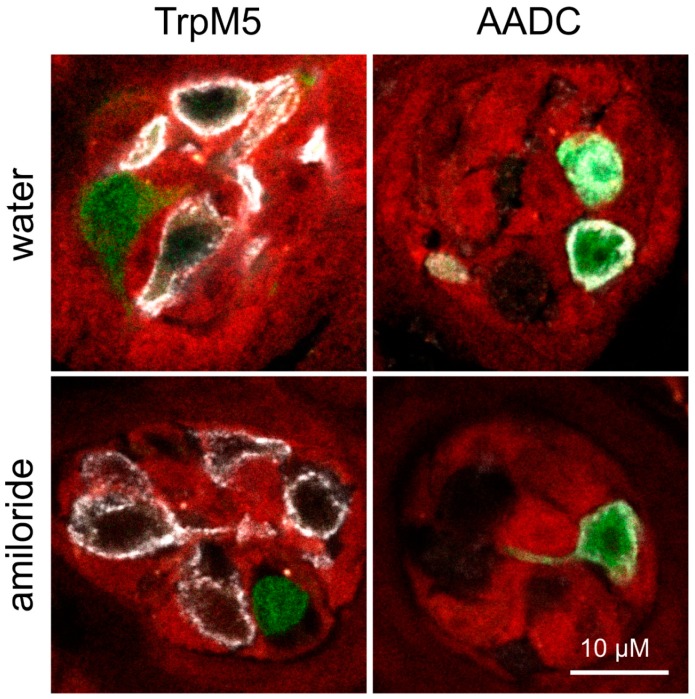
Expression of fluorescent proteins in taste papillae after amiloride intervention. Fungiform papillae sections of Scnn1^aa/bb^ animals expressing GFP (green) and tdRFP (red) fluorescence in αENaC- and βENaC-expressing cells, respectively, were stained for Type II (TrpM5) and Type III (AADC) taste cell markers after amiloride intervention. Therefore, animals received adequate salt diet without or with 300 µM amiloride-containing drinking water prior to sacrifice. Independent of intervention, GFP and tdRFP fluorescence showed no co-localization in taste papillae. Whereas GFP-positive cells always co-expressed AADC, tdRFP-positive cells revealed no overlap with the cell markers TrpM5 or AADC, visualized by immunofluorescence (white). Scale bar applies to all images.

As reported in a recent study, application of 300 µM amiloride over a period of 36 h induces robust salt appetite [38]. To investigate whether amiloride affects only the preference for sodium or also ENaC expression, Scnn1^++/++^ and Scnn1^aa/bb^ mice were subjected to short-term preference tests as well as to taste bud and tissue preparations after receiving amiloride for 36 h next to a sodium-adjusted diet. According to the latter and subsequent expression analysis, free access to amiloride-containing water did not have an impact on the relative expression values for α-, β- or γENaC subunits in non-gustatory tissue and kidney, whereas in fungiform and foliate/vallate papillae, β- and αENaC, respectively, were induced after amiloride treatment (Table 2). However, no impact of amiloride intervention on GFP and tdRFP fluorescence was recognized in fungiform papillae with regard to localization and apparent intensity (Figure 4). According to expression analysis, all 3 ENaC subunits of the distal colon were affected by amiloride intervention, with significantly higher relative expression levels after access to amiloride for 36 h (Table 2).

**Figure 5:**
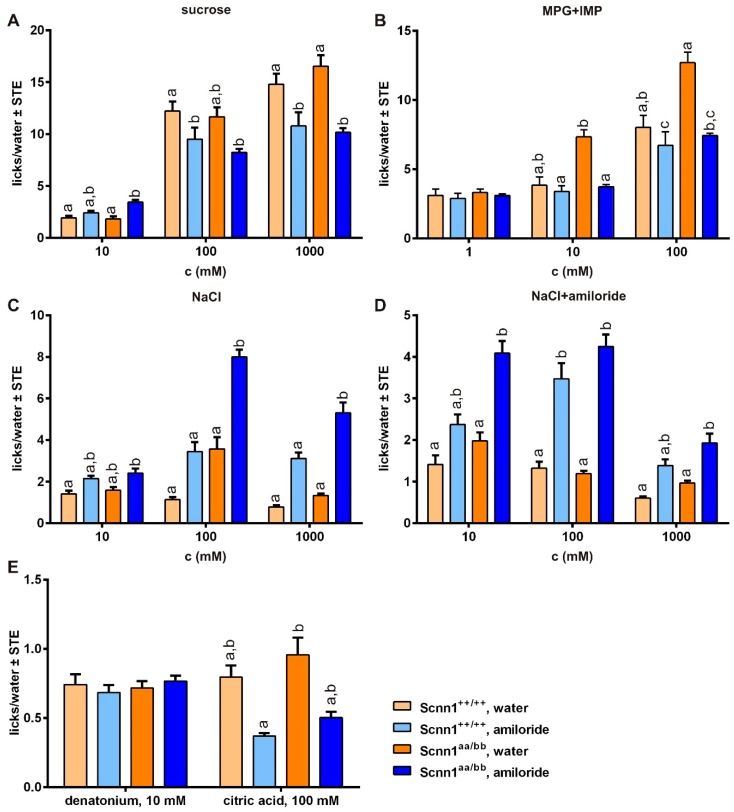
Taste responses of Scnn1^++/++^ and Scnn1^aa/bb^ mice after access to amiloride-containing water. Scnn1^++/++^ and Scnn1^aa/bb^ mice receiving a sodium-adequate diet had either access to 300 µM amiloride-containing water 13 h prior to restriction starting or received water without amiloride. The restriction phase lasted for 22.5 h with access to 2.0 mL water ± 300 µM amiloride and 1 g of food. Lick responses to different concentrated solutions of sucrose (**A**), monopotassium glutamate with inosine 5´monophosphate (MPG+IMP; **B**), sodium chloride (NaCl; **C**), NaCl with amiloride (NaCl+amiloride; **D**), or bitter and sour stimuli (**E**) were determined by an automated gustometer. Each data point represents a mean ± SE of 5 s presentations from 10 to 16 animals tested. Statistical was testing based on UNIANOVA and post-hoc analysis using Bonferroni´s multiple comparison test. Different letters indicate statistical significance.

In the short-term preference test, 3 concentrations of sucrose, MPG+IMP, NaCl, and NaCl+amiloride representing attractive taste stimuli were tested (Figure 5, Tables S4 and S5). The main differences were due to amiloride treatment. However, few additional effects of genotype were recognized (Tables S4 and S5). Accordingly, at least medium and high concentrations of sucrose and MPG+IMP resulted in significantly fewer licks after amiloride intervention (Figure 5A,B), whereas NaCl presentations led to increased licks/water ratios (Figure 5C,D). Licks of water did not show significant changes upon amiloride treatment. Aversive control stimuli (denatonium benzoate and citric acid) were only checked at a single concentration, whereas denatonium benzoate did not result in any differences between the groups and lick responses for citric acid were reduced after amiloride treatment (Figure 5E, Table S4).

In addition, amiloride treatment did not only change perceived intensity of taste solutions but also increased motivational behavior of the animals. Accordingly, amiloride-treated mice initiated more trials/completed a larger percentage of trials during the 20 min test sessions than did mice that received only water. A trial began with the opening of the shutter and ended 5 s after the mouse made its first lick on the drinking spout (see Section 2). In each session, trials were set to a maximum of 50. Whereas most of the time, animals performed about 20–30 trials per day (e.g., “water-treated” mice, Figure 5 or after dietary intervention, Figure 3), amiloride-treated animals performed about 40–50 trials per session (Figure S2). Despite considerable individual variations for all animals independent of intervention, amiloride-treated animals showed reduced latency, or the time taken to initiate the first lick of a trial, reaching statistical significance for at least one concentration of a presented stimulus. Only for concentrated control stimuli (denatonium benzoate and citric acid) was no or a converse situation recognized, however, it did not reach statistical significance (Figure S2E). Accordingly, the overall performance of amiloride-treated mice was changed.

### 3.5. Amiloride Interaction with Bitter Taste Receptors

**Figure 6:**
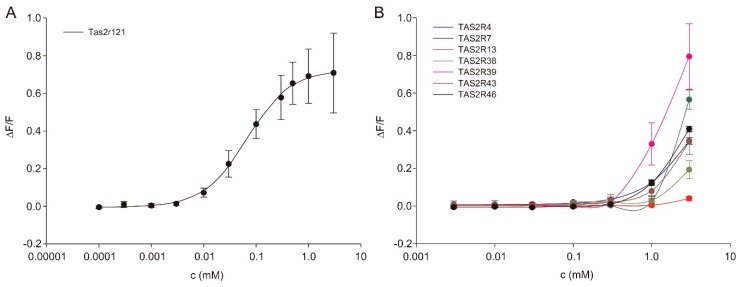
Concentration-response relations of murine (**A**) and human (**B**) bitter taste receptor-expressing cells stimulated with increasing concentrations of amiloride calculated from calcium traces acquired by fluorometric imaging plate reader (FLIPR) recordings. Changes in fluorescence (Δ*F*/*F*) were plotted semi-logarithmically versus agonist concentrations.

Amiloride was reported to be tasteless to rats and mice at or below 100 µM [41,42,43], whereas humans perceive bitterness at concentrations above 100 µM [44,45,46,47]. If, however, amiloride would also cause bitter perception in mice, this could have an unspecific impact on salt intake in case of synchronous application of amiloride. In order to confirm that amiloride could (not) activate bitter taste receptors, we performed functional expression analyses with mouse and human bitter taste receptor constructs. Transient expression of 25 human and 34 mouse bitter taste receptors revealed that amiloride indeed activated 1 and 7 bitter taste receptors, respectively (Figure 6). The activation of mouse bitter taste receptor Tas2r121 was observed at concentrations of 0.01 mM and above (Figure 6A), whereas the human bitter taste receptors, TAS2R4, TAS2R7, TAS2R13, TAS2R38, TAS2R39, TAS2R43, and TAS2R46 revealed an activation by amiloride at ~100 times higher concentrations (Figure 6B).

## 4. Discussion

Modern society is characterized by high consumption of table salt [48,49,50,51,52], which increases risks of diseases such as stroke, left ventricular hypertrophy, renal stones, or osteoporosis [53,54,55,56,57,58,59,60]. Accordingly, it is of considerable interest to elucidate and understand the molecular and cellular basis of salt taste. In 2010 the relevance of the αENaC subunit in attractive salt taste in mice was confirmed by a conditional knock-out in taste bud cells, impairing amiloride-sensitive salt taste detection, while retaining normal responses to other taste qualities as well as high salt reception [32,61]. This is in agreement with the observation that salt taste is, at least in mice, partially affected by the diuretic drug amiloride, a well-known effector of ENaC in the kidney [9,10,11,12]. Based on functional expression experiments in *Xenopus* oocytes, formation of a fully functional ENaC depends on the simultaneous presence of α-, β-, and γ-subunits, even though the α-subunit alone is sufficient to induce weak sodium currents [62,63,64,65,66]. However, a recent study with gene-targeted mice, labeling α- and βENaC-expressing cells by fluorescent proteins, revealed almost no co-localization of the different subunits in taste papillae [33]. To investigate if table salt restriction or overconsumption affects ENaC expression, we used the same α- and βENaC knock-in animals for a dietary intervention study. Over a period of 4 weeks, animals received either an adequate, low, or high salt diet with free access to food and water. Variable sodium content of the diet did not result in any changes in averaged food intake (Figure 1B), supporting the assumption that the caloric need determines the amount of food that is ingested rather than the sodium content. From the time point of dietary change, animals receiving a high salt diet showed a drastic increase in water intake, whereas sodium depletion caused no alterations in water intake in comparison to animals fed an adequate sodium diet (Figure 1C). Accordingly, only ingestion of the high salt diet provided a rapid osmotic stimulation of thirst, as stated before for rats [67,68]. While some reports observed that sodium depletion by low salt diet did not enhance dietary sodium ingestion [69,70], others reported an induction of sodium appetite [71]. Our data suggest the absence of compensatory sodium ingestion. As oral sodium consumption rapidly quenches sodium appetite [72,73], an altered hedonic valance of sodium depending on the body’s needs is assumed [71,74,75]. To measure the ‘liking’ of taste solutions, short-term preference tests were performed (Figure 3). In order to test the wide range of behavioral responses, attractive and aversive restriction conditions were applied. Under conditions favoring attraction behavior, we observed a higher mean lick ratio at 300 mM NaCl in Scnn1^aa/bb^ animals fed with adequate and low salt diet in comparison to the other groups, whereas lick ratios for NaCl in Scnn1^aa/bb^ animals fed with a high salt diet gradually decreased with NaCl concentration (Figure 3C). In comparison, Scnn1^++/++^ mice were relatively indifferent to all NaCl concentrations in comparison to water and did not show concentration-dependent changes in their lick ratio. Under water-restricted (aversive) conditions Scnn1^++/++^ and Scnn1^aa/bb^ animals fed with low salt diet as well as Scnn1^aa/bb^ animals fed with sodium-adequate diet revealed less aversive behavior towards hypertonic saline solutions (300 and 1000 mM) than animals fed with high salt diet or Scnn1^++/++^ animals fed with sodium-adequate diet (Figure 3G). Accordingly, depending on the table salt content of the diet, mice became significantly more adept at avoiding high salt-containing solutions to satisfy the demand for an adequate sodium supply. This is in line with the observation that sodium depletion does neither alter perceived intensity nor quality, assuming that only the hedonic character of table salt is affected [76]. Indeed, sodium-depleted human subjects also displayed an increased preference for high salt diets [77]. Moreover, sodium-depleted rodents drink sodium-containing solutions even at concentrations they would normally reject [18]. Accordingly, sodium deficits seem to trigger an intake behavior towards concentrated sodium solutions.

Furthermore, preference for table salt was reduced by co-application of amiloride without remaining variations between the different diet X genotype constellations under attractive restriction conditions; fitting to the observation that exposure to amiloride diminishes licks to table salt [78,79]. The co-application of amiloride and NaCl under water-restricted conditions had only modest effects on NaCl avoidance (Figure 3H), supporting the view that amiloride-insensitive pathways mediate the detection of high sodium concentrations [80,81,82,83].

Conspicuously, next to the diet, expression levels of ENaC subunits also seem to affect NaCl taste. Mice lacking Engrailed-2, a transcription factor critical for neural development, showed an increase in the expression of αENaC subunits in lingual taste papillae, accompanied by increased taste responsivity to 300 mM NaCl and reduced avoidance of salt [84]. Knock-in of fluorescent proteins in the here used Scnn1^aa/bb^ mice also resulted in higher mRNA expression levels of ENaC subunits in taste papillae, but not in non-gustatory lingual tissues (Table S1). These differences in gene expression are probably due to the endowment of both loci with bovine growth hormone polyadenylation signal (BGH) as part of the gene targeting strategy [33]. The presence of the BGH signal results in increased stability and by that mild overexpression of α- and βENaC mRNA [85,86], potentially resulting in the production of higher polypeptide levels from the recombinant locus and eventually accounting for differences between both genotypes (Tables S2 and S3). This is seen, for instance, in the case of the Scnn1^aa/bb^ knock-in mice fed with a sodium-adequate diet, who showed higher lick/water ratios for NaCl (100 to 1000 mM) than Scnn1^++/++^ mice under attractive restriction conditions (Figure 3C). In comparison to these observations, Scnn1^aa/bb^ animals fed with a low salt diet seem more susceptible to the exposure of high NaCl concentrations, resulting in higher lick ratios at 100, 300, and 1000 mM in comparison to corresponding Scnn1^++/++^ animals (Figure 3C). This effect was not seen if NaCl was accompanied by amiloride (Figure 3D). Moreover, it seems that Scnn1^aa/bb^ knock-in mice overexpressing α- and βENaC in general are more sensitive to low MPG+IMP and less sensitive towards high concentrated salt and sour stimuli.

Compared to genotype, either feeding of low or high sodium-containing diets failed to significantly alter relative RNA expression levels of ENaC subunits in gustatory tissue (Table 1). These observations confirm earlier studies, reporting unchanged transcription levels for ENaC subunits in taste buds of sodium-deprived rodents compared with animals fed a control diet [28,36,87,88]. Additionally, no changes in fluorescent protein expression (GFP and tdRFP) were observed, neither regarding cellular localization nor intensity in fungiform papillae. Accordingly, GFP fluorescence was only recognized in Type III cells, whereas tdRFP fluorescence was restricted to non-Type II and -Type III cells (Figure 2). However, with regard to physiologically potentially more relevant tissues for sodium homeostasis, elevated ENaC expression was recognized in the distal colon of animals, which were fed a low salt diet (Table 1). These results confirm and extend earlier studies which reported that low salt diet or application of aldosterone resulted in increased transcription of β- and γENaC subunits in the colon, whereas αENaC subunit transcription remained unchanged [36,89,90,91]. Some groups further reported that sodium-depleted rats not only showed altered ENaC mRNA levels, but also higher αENaC protein levels in the colon in comparison to animals fed a high salt diet [89,90]. In comparison to that, mice fed a high salt diet showed significantly reduced αENaC expression levels in the kidney in comparison to animals that received a sodium-adequate diet (Table 1). Previously, low salt diet or application of aldosterone was observed to induce the expression of the αENaC subunit in the kidney, accompanied with unchanged expression for β- and γENaC subunits [5,92,93,94,95,96], which was also not observed in this study. Accordingly, compensatory effects for maintaining sodium homeostasis are realized via the colon and kidney rather than taste tissue with regard to adaptations in ENaC expression, indicating the pivotal role of these tissues in sodium reabsorption [97,98].

In addition to dietary intervention, salt appetite in mice was reported to be induced more robustly by exposure to 300 µM amiloride in drinking water over a period of 36 h, as reported recently [38]. Access to 300 µM amiloride-containing drinking water for 36 h resulted in significantly higher expression levels for β- (and nearly also for αENaC) and αENaC in fungiform and posterior lingual papillae, respectively (Table 2). Additionally, once more in the distal colon, all ENaC subunit transcription levels were significantly increased after amiloride treatment (Table 2). Furthermore, lick responses to different taste solutions were altered (Figure 5, Tables S4 and S5). We observed increased lick ratios for NaCl potentially based on a sodium imbalance due to blocked ENaC channels (Figure 5C,D) as well as reduced lick/water ratios for most of the tested concentrations of sucrose and MPG+IMP (Figure 5A,B), indicating perceptual changes and/or reduced attractiveness to the mice. At least for humans, amiloride was recognized to suppress sweet taste [99,100,101]. Cell culture experiments using a cell line stably expressing human sweet taste receptor revealed that in the presence of 3 mM amiloride responses to sweet tastants like sugars and artificial sweeteners were reduced [99,100,101]. As sweet and umami taste share molecular and hedonic similarities, comparable effects of long-term amiloride exposure to both taste qualities appear reasonable. Moreover, a reduced lick/water ratio was recognized for 100 mM citric acid (Figure 5E). Whether interactions of amiloride with αENaC, which is expressed in sour-mediating Type III cells, or acid-sensing ion channels (ASICs) might be responsible for this, requires further analyses. At least after short amiloride exposure, neural responses to citric or hydrochloric acid were not affected by amiloride in various species [99,102,103,104], including the mouse [105]. Otherwise, preliminary results (unpublished) showed that at concentrations above 100 µM amiloride resulted in reduced lick/water ratios in short-term preference tests, indicating that aversive taste perception is triggered. Previously, amiloride has been reported to taste bitter to humans at concentrations above 100 µM [44,45,46,47], whereas amiloride was reported to be tasteless to rats and mice at or below 100 µM [41,42,43]. Our functional expression analysis now identified Tas2r121 as a murine bitter taste receptor for amiloride (Figure 6). This new finding may also have an impact on the observed species differences concerning the amiloride sensitivity of salt taste [44,46,104]. Moreover, the finding that amiloride tastes bitter to mice suggests that amiloride treatment does not only block attraction to low sodium chloride concentrations, but also represents at the same time an aversive taste stimulus. This should be kept in mind when interpreting and planning such experiments.

In summary, this study showed that sodium depletion, feeding a hypertonic saline diet, and amiloride intervention impact taste liking and ENaC expression, with differences regarding subunits and organs. Thereby, colon and kidney seem to be of greater importance to compensate imbalanced sodium homeostasis than gustatory tissue based on the monitored ENaC expression levels. However, effects of genotype were also recognized. As Scnn1^aa/bb^ animals showed higher levels of ENaC subunits (at least at cDNA level) than corresponding wild-type controls, changes of ENaC expression seem to have a prominent impact on taste liking even without dietary interventions. This needs to be addressed in detail in future studies. Additionally, we could confirm that the application of 300 µM amiloride in the drinking water is an efficient way to induce salt appetite. However, amiloride did not only alter taste sensation for salt but also for sucrose, MPG+IMP, and high concentrations of citric acid, indicating a more general influence of this drug and its bitter taste on taste perception.

## Figures and Tables

**Table 1:** Relative expression of epithelial sodium channel (ENaC) subunits in Scnn1^aa/bb^ mice after dietary intervention. Based on quantitative RT-PCR, the relative expression levels of ENaC subunits normalized to the housekeeping genes β-actin and eEf2 in isolated taste buds and non-gustatory tissue were determined. Data represent the means of 6 Scnn1^aa/bb^ mice, fed with sodium-adequate, low, or high salt diet over a period of 4 weeks. Statistical testing is based on UNIANOVA and post-hoc analysis using Bonferroni´s multiple comparison test. *p*-Values rely on comparison of all groups with significant differences indicated in bold; individual differences between the different diets are additionally indicated as the following: * statistical significance between sodium-adequate and high salt diet; ^#^ statistical significance between sodium-adequate and low salt diet; ^$^ statistical significance between low and high salt diet based on Student´s *t*-test.

	ENaC Subunit	Adequate (mean ± STABW)	Low Salt (mean ± STABW)	High Salt (mean ± STABW)	*p*-Value
fungiform papillae	α	0.0151 ± 0.0053	0.0174 ± 0.0098	0.0201 ± 0.0104	0.735
	β	0.0062 ± 0.0030	0.0101 ± 0.0059	0.0087 ± 0.0069	0.464
	γ	0.0093 ± 0.0026	0.0118 ± 0.0066	0.0117 ± 0.0075	0.719
vallate and foliate papillae	α	0.0606 ± 0.0111	0.0577 ± 0.0217	0.0538 ± 0.0152	0.780
	β	0.0027 ± 0.0007	0.0024 ± 0.0010	0.0036 ± 0.0009	0.084
	γ	0.0020 ± 0.0005	0.0018 ± 0.0007	0.0021 ± 0.0004	0.669
non-gustatory epithelium	α	0.0228 ± 0.0061	0.0283 ± 0.0107	0.0205 ± 0.0037	0.212
	β	0.0011 ± 0.0004	0.0010 ± 0.0003	0.0010 ± 0.0002	0.942
	γ	0.0008 ± 0.0001	0.0007 ± 0.0003	0.0007 ± 0.0002	0.891
kidney	α	0.0698 ± 0.0235	0.0600 ± 0.0154	0.0420 ± 0.0069	**0.034** *
	β	0.0260 ± 0.0086	0.0196 ± 0.0043	0.0215 ± 0.0043	0.211
	γ	0.0425 ± 0.0112	0.0296 ± 0.0036	0.0360 ± 0.0084	0.054
distal colon	α	0.0357 ± 0.0104	0.0504 ± 0.0102	0.0266 ± 0.0063	**0.002** ^#,$^
	β	0.0051 ± 0.0017	0.0258 ± 0.0184	0.0026 ± 0.0010	**0.003** ^#,$^
	γ	0.0091 ± 0.0068	0.0306 ± 0.0302	0.0027 ± 0.0026	**0.041** ^$^

**Table 2:** Relative expression of ENaC subunits in Scnn1^aa/bb^ mice after amiloride intervention. Based on quantitative RT-PCR the relative expression levels of ENaC subunits normalized to the housekeeping genes β-actin and eEf2 in isolated taste buds and non-gustatory tissue were determined. Data represent means of 6 Scnn1^aa/bb^ mice, with access to a sodium-adequate diet and water or 300 µM amiloride-containing drinking solution for 36 h prior to tissue isolation. Statistical testing was based on Student´s *t*-test. Differences were considered to be significant if *p* < 0.05, as indicated in bold.

	ENaC Subunit	Water (mean ± STABW)	Amiloride (mean ± STABW)	*p*-Value
fungiform papillae	α	0.0104 ± 0.0019	0.0136 ± 0.0019	0.051
	β	0.0030 ± 0.0014	0.0060 ± 0.0012	**0.017**
	γ	0.0056 ± 0.0025	0.0063 ± 0.0024	0.700
vallate and foliate papillae	α	0.0529 ± 0.0089	0.0700 ± 0.0083	**0.031**
	β	0.0024 ± 0.0003	0.0028 ± 0.0006	0.311
	γ	0.0018 ± 0.0004	0.0017 ± 0.0003	0.853
non-gustatory epithelium	α	0.0229 ± 0.0041	0.0280 ± 0.0019	0.066
	β	0.0008 ± 0.0004	0.0013 ± 0.0004	0.195
	γ	0.0007 ± 0.0002	0.0008 ± 0.0003	0.682
kidney	α	0.0583 ± 0.0217	0.0744 ± 0.0199	0.317
	β	0.0223 ± 0.0061	0.0238 ± 0.0057	0.738
	γ	0.0399 ± 0.0172	0.0326 ± 0.0034	0.432
distal colon	α	0.0256 ± 0.0097	0.0597 ± 0.0164	**0.012**
	β	0.0051 ± 0.0018	0.0428 ± 0.0105	**<0.001**
	γ	0.0067 ± 0.0038	0.0684 ± 0.0227	**0.002**

## Supplementary Materials

The following are available online at https://www.mdpi.com/2072-6643/12/4/995/s1, Figure S1: Taste responses of Scnn1^++/++^ and Scnn1^aa/bb^ mice to control stimuli after dietary intervention, Figure S2: Latency to initiate the first lick for different taste stimuli after access to amiloride-containing water, Table S1: Relative expression of ENaC subunits in Scnn1^++/++^ and Scnn1^aa/bb^ mice, Table S2: Statistical significance of different factors on the short-term preference tests of Scnn1^++/++^ and Scnn1^aa/bb^ animals after dietary intervention, Table S3: Statistical significance of different factors on the short-term preference tests of Scnn1^++/++^ and Scnn1^aa/bb^ animals, Table S4: Statistical significance of different factors on the short-term preference tests of Scnn1^++/++^ and Scnn1^aa/bb^ animals after amiloride intervention for 36 h, Table S5: Statistical significance of different factors on the short-term preference tests of Scnn1^++/++^ and Scnn1^aa/bb^ animals after access to amiloride-containing drinking water for 36 h.

## References

[1] Hollenberg N.K. (1982). Surfeit, deficit, and the set point for sodium homeostasis. Kidney Int..

[2] Damkjaer M., Jensen P.H., Schwammle V., Sprenger R.R., Jacobsen I.A., Jensen O.N., Bie P. (2014). Selective renal vasoconstriction, exaggerated natriuresis and excretion rates of exosomic proteins in essential hypertension. Acta Physiol. (Oxf.).

[3] Healy V., Thompson C., Johns E.J. (2014). The adrenergic regulation of proximal tubular Na(+)/H(+) exchanger 3 in the rat. Acta Physiol. (Oxf.).

[4] Fujita T. (2008). Aldosterone in salt-sensitive hypertension and metabolic syndrome. J. Mol. Med. (Berl.).

[5] Loffing J., Pietri L., Aregger F., Bloch-Faure M., Ziegler U., Meneton P., Rossier B.C., Kaissling B. (2000). Differential subcellular localization of ENaC subunits in mouse kidney in response to high- and low-Na diets. Am. J. Physiol. Ren. Physiol..

[6] Duc C., Farman N., Canessa C.M., Bonvalet J.P., Rossier B.C. (1994). Cell-specific expression of epithelial sodium channel alpha, beta, and gamma subunits in aldosterone-responsive epithelia from the rat: Localization by in situ hybridization and immunocytochemistry. J. Cell Biol..

[7] Kunzelmann K., Mall M. (2002). Electrolyte transport in the mammalian colon: Mechanisms and implications for disease. Physiol. Rev..

[8] Koyama K., Sasaki I., Naito H., Funayama Y., Fukushima K., Unno M., Matsuno S., Hayashi H., Suzuki Y. (1999). Induction of epithelial Na+ channel in rat ileum after proctocolectomy. Am. J. Physiol..

[9] Heck G.L., Mierson S., DeSimone J.A. (1984). Salt taste transduction occurs through an amiloride-sensitive sodium transport pathway. Science.

[10] Brand J.G., Teeter J.H., Silver W.L. (1985). Inhibition by amiloride of chorda tympani responses evoked by monovalent salts. Brain Res..

[11] Garty H., Palmer L.G. (1997). Epithelial sodium channels: Function, structure, and regulation. Physiol. Rev..

[12] Kashlan O.B., Kleyman T.R. (2011). ENaC structure and function in the wake of a resolved structure of a family member. Am. J. Physiol. Ren. Physiol..

[13] Cragoe E.J., Woltersdorf O.W., Bicking J.B., Kwong S.F., Jones J.H. (1967). Pyrazine diuretics. II. N-amidino-3-amino-5-substituted 6-halopyrazinecarboxamides. J. Med. Chem..

[14] Cohen A.B. (1966). Hyperkalemic effects of triamterene. Ann. Intern. Med..

[15] Gombos E.A., Freis E.D., Moghadam A. (1966). Effects of MK-870 in normal subjects and hypertensive patients. N. Engl. J. Med..

[16] Saha C., Eckert G.J., Ambrosius W.T., Chun T.Y., Wagner M.A., Zhao Q., Pratt J.H. (2005). Improvement in blood pressure with inhibition of the epithelial sodium channel in blacks with hypertension. Hypertension.

[17] Denton D.A. (1882). The Hunger for Salt.

[18] St John S.J. (2017). The perceptual characteristics of sodium chloride to sodium-depleted rats. Chem. Senses.

[19] Geerling J.C., Loewy A.D. (2008). Central regulation of sodium appetite. Exp. Physiol..

[20] Johnson A.K., Thunhorst R.L. (1997). The neuroendocrinology of thirst and salt appetite: Visceral sensory signals and mechanisms of central integration. Front. Neuroendocrinol..

[21] Fischer N.M., Schiefer C.P., St John S.J. (2016). Gustatory contributions to sodium appetite: A microstructural analysis. Chem. Senses.

[22] Sato T., Miyamoto T., Okada Y. (1994). Comparison of gustatory transduction mechanisms in vertebrate taste cells. Zool. Sci..

[23] Lindemann B. (1996). Taste reception. Physiol. Rev..

[24] Stewart R.E., DeSimone J.A., Hill D.L. (1997). New perspectives in a gustatory physiology: Transduction, development, and plasticity. Am. J. Physiol..

[25] Benos D.J. (1982). Amiloride: A molecular probe of sodium transport in tissues and cells. Am. J. Physiol..

[26] Lindemann B. (2001). Receptors and transduction in taste. Nature.

[27] Ninomiya Y., Funakoshi M. (1988). Amiloride inhibition of responses of rat single chorda tympani fibers to chemical and electrical tongue stimulations. Brain Res..

[28] Lin W., Finger T.E., Rossier B.C., Kinnamon S.C. (1999). Epithelial Na+ channel subunits in rat taste cells: Localization and regulation by aldosterone. J. Comp. Neurol..

[29] Gilbertson T.A., Kinnamon S.C. (1996). Making sense of chemicals. Chem. Biol..

[30] Boughter J.D., Gilbertson T.A. (1999). From channels to behavior: An integrative model of NaCl taste. Neuron.

[31] Kretz O., Barbry P., Bock R., Lindemann B. (1999). Differential expression of RNA and protein of the three pore-forming subunits of the amiloride-sensitive epithelial sodium channel in taste buds of the rat. J. Histochem. Cytochem. Off. J. Histochem. Soc..

[32] Chandrashekar J., Kuhn C., Oka Y., Yarmolinsky D.A., Hummler E., Ryba N.J., Zuker C.S. (2010). The cells and peripheral representation of sodium taste in mice. Nature.

[33] Lossow K., Hermans-Borgmeyer I., Meyerhof W., Behrens M. (2020). Segregated expression of ENaC subunits in taste cells. Chem. Senses.

[34] Jasti J., Furukawa H., Gonzales E.B., Gouaux E. (2007). Structure of acid-sensing ion channel 1 at 1.9 A resolution and low pH. Nature.

[35] Noreng S., Bharadwaj A., Posert R., Yoshioka C., Baconguis I. (2018). Structure of the human epithelial sodium channel by cryo-electron microscopy. Elife.

[36] Huang T., Stahler F. (2009). Effects of dietary Na+ deprivation on epithelial Na+ channel (ENaC), BDNF, and TrkB mRNA expression in the rat tongue. Bmc Neurosci..

[37] Hedrich H. (2004). The Laboratory Mouse.

[38] Raymond M.A., Chowdhury T., Mast T.G., Breza J.M. (2017). A simple method for a rapid induction of salt appetite in mice. Annu. Meet. Assoc. Chemorecept. Sci..

[39] Lossow K., Hubner S., Roudnitzky N., Slack J.P., Pollastro F., Behrens M., Meyerhof W. (2016). Comprehensive analysis of mouse bitter taste receptors reveals different molecular receptive ranges for orthologous receptors in mice and humans. J. Biol. Chem..

[40] Kusumakshi S., Voigt A., Hubner S., Hermans-Borgmeyer I., Ortalli A., Pyrski M., Dorr J., Zufall F., Flockerzi V., Meyerhof W. (2015). A binary genetic approach to characterize trpm5 cells in mice. Chem. Senses.

[41] Hill D.L., Formaker B.K., White K.S. (1990). Perceptual characteristics of the amiloride-suppressed sodium chloride taste response in the rat. Behav. Neurosci..

[42] Markison S., Spector A.C. (1995). Amiloride is an ineffective conditioned stimulus in taste aversion learning. Chem. Senses.

[43] Eylam S., Tracy T., Garcea M., Spector A.C. (2003). Amiloride is an ineffective conditioned stimulus in taste aversion learning in C57BL/6J and DBA/2J mice. Chem. Senses.

[44] Desor J.A., Finn J. (1989). Effects of amiloride on salt taste in humans. Chem. Senses.

[45] McCutcheon N.B. (1992). Human psychophysical studies of saltiness suppression by amiloride. Physiol. Behav..

[46] Smith D.V., Ossebaard C.A. (1995). Amiloride suppression of the taste intensity of sodium chloride: Evidence from direct magnitude scaling. Physiol. Behav..

[47] Halpern B.P. (1998). Amiloride and vertebrate gustatory responses to NaCl. Neurosci. Biobehav. Rev..

[48] Brown I.J., Tzoulaki I., Candeias V., Elliott P. (2009). Salt intakes around the world: Implications for public health. Int. J. Epidemiol..

[49] Mozaffarian D., Fahimi S., Singh G.M., Micha R., Khatibzadeh S., Engell R.E., Lim S., Danaei G., Ezzati M., Powles J. (2014). Global sodium consumption and death from cardiovascular causes. N. Engl. J. Med..

[50] O′Donnell M., Mente A., Rangarajan S., McQueen M.J., Wang X., Liu L., Yan H., Lee S.F., Mony P., Devanath A. (2014). Urinary sodium and potassium excretion, mortality, and cardiovascular events. N. Engl. J. Med..

[51] WHO (2012). Guideline: Sodium Intake for Adults and Children.

[52] Meneton P., Jeunemaitre X., de Wardener H.E., MacGregor G.A. (2005). Links between dietary salt intake, renal salt handling, blood pressure, and cardiovascular diseases. Physiol. Rev..

[53] He F.J., MacGregor G.A. (2010). Reducing population salt intake worldwide: From evidence to implementation. Prog. Cardiovasc. Dis..

[54] He F.J., MacGregor G.A. (2009). A comprehensive review on salt and health and current experience of worldwide salt reduction programmes. J. Hum. Hypertens..

[55] Bibbins-Domingo K., Chertow G.M., Coxson P.G., Moran A., Lightwood J.M., Pletcher M.J., Goldman L. (2010). Projected effect of dietary salt reductions on future cardiovascular disease. N. Engl. J. Med..

[56] Sacks F.M., Campos H. (2010). Dietary therapy in hypertension. N. Engl. J. Med..

[57] Tsugane S., Sasazuki S., Kobayashi M., Sasaki S. (2004). Salt and salted food intake and subsequent risk of gastric cancer among middle-aged Japanese men and women. Br. J. Cancer.

[58] Devine A., Criddle R.A., Dick I.M., Kerr D.A., Prince R.L. (1995). A longitudinal study of the effect of sodium and calcium intakes on regional bone density in postmenopausal women. Am. J. Clin. Nutr..

[59] Asaria P., Chisholm D., Mathers C., Ezzati M., Beaglehole R. (2007). Chronic disease prevention: Health effects and financial costs of strategies to reduce salt intake and control tobacco use. Lancet.

[60] Strazzullo P., D′Elia L., Kandala N.B., Cappuccio F.P. (2009). Salt intake, stroke, and cardiovascular disease: Meta-analysis of prospective studies. BMJ.

[61] Bosak N.P., Inoue M., Nelson T.M., Hummler E., Ishiwatari Y., Bachmanov A.A. (2010). Epithelial sodium channel (ENaC) is involved in reception of sodium taste: Evidence from mice with a tissue-specific conditional targeted mutation of the ENaC gene (abstract), thirty-second annual meeting of the association for chemoreception sciences. Chem. Senses.

[62] Canessa C.M., Schild L., Buell G., Thorens B., Gautschi I., Horisberger J.D., Rossier B.C. (1994). Amiloride-sensitive epithelial Na+ channel is made of three homologous subunits. Nature.

[63] McDonald F.J., Price M.P., Snyder P.M., Welsh M.J. (1995). Cloning and expression of the beta- and gamma-subunits of the human epithelial sodium channel. Am. J. Physiol..

[64] Giraldez T., Afonso-Oramas D., Cruz-Muros I., Garcia-Marin V., Pagel P., Gonzalez-Hernandez T., Alvarez de la Rosa D. (2007). Cloning and functional expression of a new epithelial sodium channel delta subunit isoform differentially expressed in neurons of the human and monkey telencephalon. J. Neurochem..

[65] Edelheit O., Hanukoglu I., Dascal N., Hanukoglu A. (2011). Identification of the roles of conserved charged residues in the extracellular domain of an epithelial sodium channel (ENaC) subunit by alanine mutagenesis. Am. J. Physiol. Ren. Physiol..

[66] Edelheit O., Ben-Shahar R., Dascal N., Hanukoglu A., Hanukoglu I. (2014). Conserved charged residues at the surface and interface of epithelial sodium channel subunits--roles in cell surface expression and the sodium self-inhibition response. Febs J..

[67] Stricker E.M., Hoffmann M.L., Riccardi C.J., Smith J.C. (2003). Increased water intake by rats maintained on high NaCl diet: Analysis of ingestive behavior. Physiol. Behav..

[68] Ramsey D.J., Thrasher T.N., Stricker E.M. (1990). Thirst and water balance. Neurobiology of food and fluid intake. Handbook of Behavioral Neurobiology.

[69] Contreras R.J., Hatton G.I. (1975). Gustatory adaptation as an explanation for dietary-induced sodium appetite. Physiol. Behav..

[70] Stricker E.M., Thiels E., Verbalis J.G. (1991). Sodium appetite in rats after prolonged dietary sodium deprivation: A sexually dimorphic phenomenon. Am. J. Physiol..

[71] Berridge K.C., Flynn F.W., Schulkin J., Grill H.J. (1984). Sodium depletion enhances salt palatability in rats. Behav. Neurosci..

[72] Nachman M., Valentino D.A. (1966). Roles of taste and postingestional factors in the satiation of sodium appetite in rats. J. Comp. Physiol. Psychol..

[73] Wolf G., Schulkin J., Simson P.E. (1984). Multiple factors in the satiation of salt appetite. Behav. Neurosci..

[74] Handal P.J. (1965). Immediate acceptance of sodium salts by sodium deficient rats. Psychon. Sci..

[75] Fortin S.M., Roitman M.F. (2017). Physiological state tunes mesolimbic signaling: Lessons from sodium appetite and inspiration from Randall R. Sakai. Physiol. Behav..

[76] St John S.J., McBrayer A.M., Krauskopf E.E. (2017). Sodium carbonate is saltier than sodium chloride to sodium-depleted rats. Chem. Senses.

[77] Beauchamp G.K., Bertino M., Burke D., Engelman K. (1990). Experimental sodium depletion and salt taste in normal human volunteers. Am. J. Clin. Nutr..

[78] Bernstein I.L., Hennessy C.J. (1987). Amiloride-sensitive sodium channels and expression of sodium appetite in rats. Am. J. Physiol..

[79] McCutcheon N.B. (1991). Sodium deficient rats are unmotivated by sodium chloride solutions mixed with the sodium channel blocker amiloride. Behav. Neurosci..

[80] Oka Y., Butnaru M., von Buchholtz L., Ryba N.J., Zuker C.S. (2013). High salt recruits aversive taste pathways. Nature.

[81] Ren Z., Rhyu M.R., Phan T.H., Mummalaneni S., Murthy K.S., Grider J.R., DeSimone J.A., Lyall V. (2013). TRPM5-dependent amiloride- and benzamil-insensitive NaCl chorda tympani taste nerve response. Am. J. Physiol. Gastrointest. Liver Physiol..

[82] Lewandowski B.C., Sukumaran S.K., Margolskee R.F., Bachmanov A.A. (2016). Amiloride-insensitive salt taste is mediated by two populations of type iii taste cells with distinct transduction mechanisms. J. Neurosci. Off. J. Soc. Neurosci..

[83] Zhang Y., Hoon M.A., Chandrashekar J., Mueller K.L., Cook B., Wu D., Zuker C.S., Ryba N.J. (2003). Coding of sweet, bitter, and umami tastes: Different receptor cells sharing similar signaling pathways. Cell.

[84] Gupta A., Li X., DiCicco-Bloom E., Bello N.T. (2018). Altered salt taste response and increased tongue epithelium Scnna1 expression in adult Engrailed-2 null mice. Physiol. Behav..

[85] Ribeiro S.C., Monteiro G.A., Prazeres D.M. (2004). The role of polyadenylation signal secondary structures on the resistance of plasmid vectors to nucleases. J. Gene Med..

[86] Azzoni A.R., Ribeiro S.C., Monteiro G.A., Prazeres D.M. (2007). The impact of polyadenylation signals on plasmid nuclease-resistance and transgene expression. J. Gene Med..

[87] Lindemann B., Barbry P., Kretz O., Bock R. (1998). Occurrence of ENaC subunit mRNA and immunocytochemistry of the channel subunits in taste buds of the rat vallate papilla. Ann. N. Y. Acad. Sci..

[88] Shigemura N., Islam A.A., Sadamitsu C., Yoshida R., Yasumatsu K., Ninomiya Y. (2005). Expression of amiloride-sensitive epithelial sodium channels in mouse taste cells after chorda tympani nerve crush. Chem. Senses.

[89] Lingueglia E., Renard S., Waldmann R., Voilley N., Champigny G., Plass H., Lazdunski M., Barbry P. (1994). Different homologous subunits of the amiloride-sensitive Na+ channel are differently regulated by aldosterone. J. Biol. Chem..

[90] Renard S., Voilley N., Bassilana F., Lazdunski M., Barbry P. (1995). Localization and regulation by steroids of the alpha, beta and gamma subunits of the amiloride-sensitive Na+ channel in colon, lung and kidney. Pflug. Arch. Eur. J. Physiol..

[91] Asher C., Wald H., Rossier B.C., Garty H. (1996). Aldosterone-induced increase in the abundance of Na+ channel subunits. Am. J. Physiol..

[92] Ono S., Kusano E., Muto S., Ando Y., Asano Y. (1997). A low-Na+ diet enhances expression of mRNA for epithelial Na+ channel in rat renal inner medulla. Pflug. Arch. Eur. J. Physiol..

[93] Stokes J.B., Sigmund R.D. (1998). Regulation of rENaC mRNA by dietary NaCl and steroids: Organ, tissue, and steroid heterogeneity. Am. J. Physiol..

[94] Masilamani S., Kim G.H., Mitchell C., Wade J.B., Knepper M.A. (1999). Aldosterone-mediated regulation of ENaC alpha, beta, and gamma subunit proteins in rat kidney. J. Clin. Investig..

[95] MacDonald P., MacKenzie S., Ramage L.E., Seckl J.R., Brown R.W. (2000). Corticosteroid regulation of amiloride-sensitive sodium-channel subunit mRNA expression in mouse kidney. J. Endocrinol..

[96] Frindt G., Palmer L.G. (2012). Regulation of epithelial Na+ channels by adrenal steroids: Mineralocorticoid and glucocorticoid effects. Am. J. Physiol. Ren. Physiol..

[97] Malsure S., Wang Q., Charles R.P., Sergi C., Perrier R., Christensen B.M., Maillard M., Rossier B.C., Hummler E. (2014). Colon-specific deletion of epithelial sodium channel causes sodium loss and aldosterone resistance. J. Am. Soc. Nephrol. JASN.

[98] Marunaka Y., Marunaka R., Sun H., Yamamoto T., Kanamura N., Taruno A. (2016). Na(+) homeostasis by epithelial Na(+) channel (ENaC) and Nax channel (Nax): Cooperation of ENaC and Nax. Ann. Transl. Med..

[99] Schiffman S.S., Lockhead E., Maes F.W. (1983). Amiloride reduces the taste intensity of Na+ and Li+ salts and sweeteners. Proc. Natl. Acad. Sci. USA.

[100] Imada T., Misaka T., Fujiwara S., Okada S., Fukuda Y., Abe K. (2010). Amiloride reduces the sweet taste intensity by inhibiting the human sweet taste receptor. Biochem. Biophys. Res. Commun..

[101] Yee K.K., Sukumaran S.K., Kotha R., Gilbertson T.A., Margolskee R.F. (2011). Glucose transporters and ATP-gated K+ (KATP) metabolic sensors are present in type 1 taste receptor 3 (T1r3)-expressing taste cells. Proc. Natl. Acad. Sci. USA.

[102] Hellekant G., DuBois G.E., Roberts T.W., van der Wel H. (1988). On the gustatory effect of amiloride in the monkey (Macaca mulatto) *Chem*. Senses.

[103] DeSimone J.A., Callaham E.M., Heck G.L. (1995). Chorda tympani taste response of rat to hydrochloric acid subject to voltage-clamped lingual receptive field. Am. J. Physiol..

[104] Ossebaard C.A., Smith D.V. (1995). Effect of amiloride on the taste of NaCl, Na-gluconate and KCl in humans: Implications for Na+ receptor mechanisms. Chem. Senses.

[105] Miyamoto T., Fujiyama R., Okada Y., Sato T. (1998). Sour transduction involves activation of NPPB-sensitive conductance in mouse taste cells. J. Neurophysiol..

