# Supplementary material for: Sodium Imbalance in Mice Results Primarily in Compensatory Gene Regulatory Responses in Kidney and Colon, but Not in Taste Tissue
Source: Nutrients. 2020 Apr 3;12(4):995. doi: 10.3390/nu12040995 (PMC7230584; doi:10.3390/nu12040995)
Supplement: Supplementary file 1 [file nutrients-12-00995-s001.pdf]

nutrients

MDPI

Supplementary

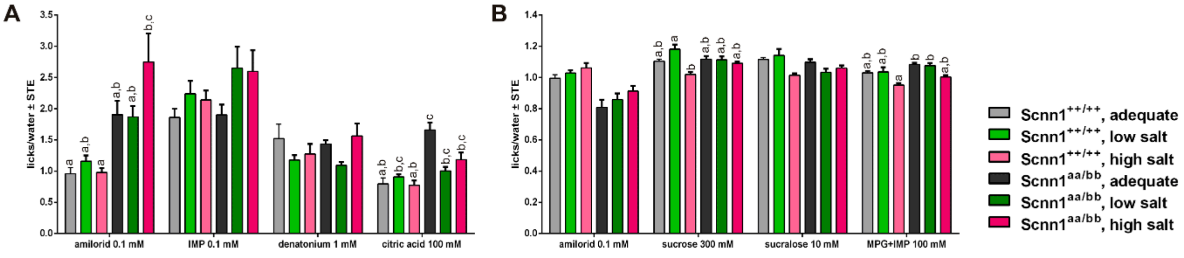

Figure S1. Taste responses of $Scnn1^{++/++}$ and $Scnn1^{aa/bb}$ mice to control stimuli after dietary intervention. After 4 weeks fed with sodium-adequate, low, or high salt diet, $Scnn1^{++/++}$ and $Scnn1^{aa/bb}$ mice were subjected to short-term preference tests using an automated gustometer. To do so, animals were either restricted for 22.5 h with access to 2.0 mL water and 1 g food (attractive restriction conditions, (**A**)) or water-deprived for 22.5 h (aversive restriction conditions, (**B**)). Taste solutions and concentrations were presented in random order. Each data point represents a mean ± STE of 5 s presentations from 10 to 11 animals tested. Statistical testing was based on UNIANOVA and post-hoc analysis using Bonferroni´s multiple comparison test. Statistical differences were indicated by different letters between individual groups.

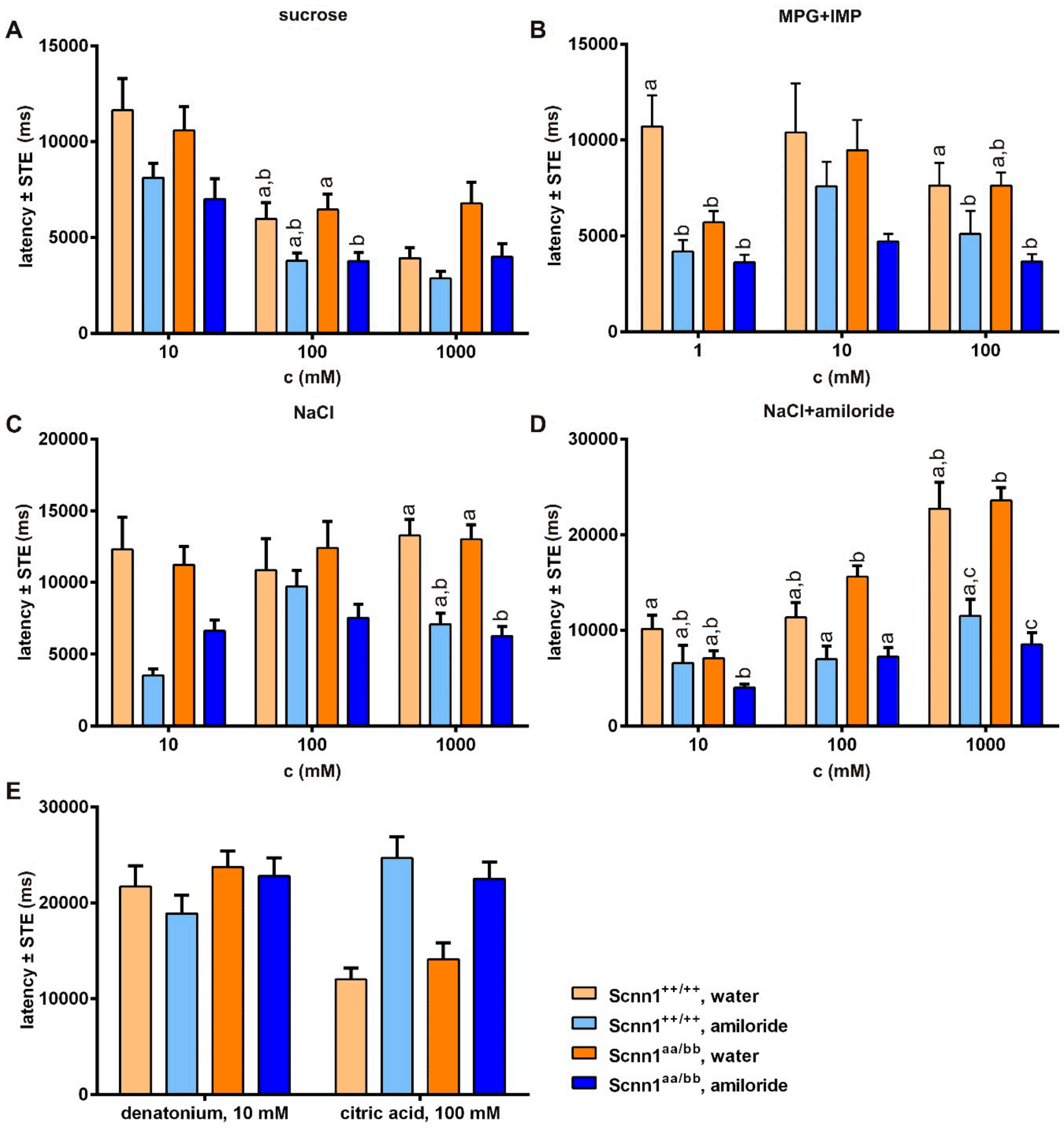

Figure S2. Latency to initiate the first lick for different taste stimuli after access to amiloride-containing water. Scnn1$^{++/++}$ and Scnn1$^{aa/bb}$ mice receiving sodium-adequate diet had either access to 300 µM amiloride-containing water 13 h prior to restriction starting or received water without amiloride. The restriction phase lasted for 22.5 h with access to 2.0 mL water ± 300 µM amiloride and 1 g food. Mean latency to the first lick for each stimulus concentration was determined by an automated gustometer presenting different concentrated solutions of sucrose (**A**), monopotassium glutamate with inosine 5′monophosphate (MPG+IMP; **B**), sodium chloride (NaCl; **C**), NaCl with amiloride (NaCl+amiloride; **D**), or bitter and sour stimuli (**E**). Each bar represents the mean ± STE from 10 to 16 animals tested. Statistical testing was based on UNIANOVA and post-hoc analysis using Bonferroni′s multiple comparison test. Different letters indicate statistical significance.

Table S1. Relative expression of ENaC subunits in Scnn1$^{++/++}$ and Scnn1$^{aa/bb}$ mice. Data represent the relative expression of ENaC subunits normalized to β-actin and eEf2 in isolated taste buds and non-gustatory tissue of Scnn1$^{++/++}$ (n = 4) and Scnn1$^{aa/bb}$ (n = 6) mice fed with a sodium-adequate diet. Mean variances between Scnn1$^{++/++}$ and Scnn1$^{aa/bb}$ animals are indicated in %. Statistical testing was based on Student´s *t*-test. Differences were considered to be significant if $p < 0.05$, as indicated in bold.

	**ENaC subunit**	**Scnn1$^{++/++}$ [33] (mean ± STABW)**	**Scnn1$^{aa/bb}$ (mean ± STABW)**	**Scnn1$^{aa/bb}$ / Scnn1$^{++/++}$ (%)**	***p*-Value**
fuP	α	0.0102 ± 0.0015	0.0151 ± 0.0053	148	0.071
	β	0.0025 ± 0.0009	0.0062 ± 0.0030	247	**0.049**
	γ	0.0044 ± 0.0018	0.0093 ± 0.0026	212	**0.011**
CV+foP	α	0.0390 ± 0.0054	0.0606 ± 0.0111	155	**0.007**
	β	0.0015 ± 0.0002	0.0027 ± 0.0007	174	**0.011**
	γ	0.0012 ± 0.0002	0.0020 ± 0.0005	166	**0.015**
non-gustatory epithelium	α	0.0208 ± 0.0032	0.0228 ± 0.0061	110	0.567
	β	0.0007 ± 0.0002	0.0011 ± 0.0004	147	0.150
	γ	0.0008 ± 0.0001	0.0008 ± 0.0001	95.0	0.623
kidney	α	0.0341 ± 0.0005	0.0698 ± 0.0235	204	**0.018**
	β	0.0157 ± 0.0026	0.0260 ± 0.0086	166	0.052
	γ	0.0278 ± 0.0145	0.0425 ± 0.0112	153	0.107
distal colon	α	0.0268 ± 0.0085	0.0357 ± 0.0104	133	0.195
	β	0.0026 ± 0.0013	0.0051 ± 0.0017	193	**0.045**
	γ	0.0052 ± 0.0054	0.0091 ± 0.0068	173	0.374

Table S2. Statistical significance of different factors on the short-term preference tests of Scnn1[++/++] and Scnn1[aa/bb] animals after dietary intervention. After 4 weeks fed with sodium-adequate, low, or high salt diet, 10 to 11 Scnn1[++/++] and Scnn1[aa/bb] mice were subjected to short-term preference tests using an automated gustometer. To do so, animals were either restricted for 22.5 h with access to 2.0 mL water and 1 g food (attractive restriction conditions) or water-deprived for 22.5 h (aversive restriction conditions). Statistical significance (*p*-value) of lick responses to different concentrations of taste solutions based on diet (sodium-adequate, low, or high), genotype (Scnn1[++/++] versus Scnn1[aa/bb]), and diet X genotype interactions are shown. Statistical testing was based on UNIANOVA and post-hoc analysis using Bonferroni´s multiple comparison test. Differences were considered to be statistically significant if $p < 0.05$, as indicated in bold.

Protocol	Substance	Concentration	Diet	Genotype	Diet X genotype
attractive	sucrose	10	0.736	0.761	0.609
		30	**0.022**	**0.021**	**<0.001**
		100	0.182	0.412	0.376
		300	0.461	0.784	0.842
		1000	0.479	0.176	0.151
	MPG+IMP	1	0.282	**<0.001**	**<0.001**
		3	**0.013**	**0.032**	**0.002**
		10	0.957	**<0.001**	**<0.001**
		30	0.914	0.824	0.962
		100	0.166	0.985	0.545
	NaCl	10	0.140	0.214	0.089
		30	**0.009**	0.122	**0.014**
		100	0.739	**<0.001**	**0.029**
		300	0.303	**0.034**	**0.042**
		1000	0.359	**0.004**	**0.049**
	NaCl+amiloride	10	0.250	0.058	0.125
		30	0.599	0.127	0.431
		100	0.352	0.204	0.297
		300	0.430	0.078	0.205
		1000	0.596	0.490	0.548
	amiloride	0.1	0.414	**0.001**	**0.012**
	IMP	0.1	0.380	0.457	0.724
	denatonium	1	0.293	0.663	0.764
	citric aid	100	0.113	**0.006**	**0.009**
aversive	denatonium	0.1	0.460	**0.002**	**0.006**
		0.3	0.545	**0.001**	**0.035**
		1	0.308	**0.030**	**0.038**
		3	0.893	0.256	0.662
		10	0.914	0.240	0.884
		1	0.124	**<0.001**	**0.003**

Protocol	Substance	Concentration	Diet	Genotype	Diet X genotype
	citric acid	3	0.380	0.963	0.364
		10	0.362	**<0.001**	**<0.001**
		30	0.209	**0.045**	0.105
		100	**0.005**	**0.022**	**0.003**
	NaCl	10	0.171	0.081	0.090
		30	0.861	0.632	0.759
		100	0.183	0.505	0.525
		300	**0.002**	0.050	**<0.001**
		1000	**<0.001**	**<0.001**	**<0.001**
	NaCl+amiloride	10	0.068	0.754	**0.035**
		30	**0.002**	0.144	**<0.001**
		100	0.067	0.307	0.284
		300	**0.002**	0.354	**0.007**
		1000	**0.001**	**0.001**	**<0.001**
	amiloride	0.1	0.389	**0.002**	**0.028**
	sucrose	300	**0.009**	0.815	**0.007**
	sucralose	10	0.080	0.166	**0.025**
	MPG+IMP	100	**0.001**	**0.004**	**<0.001**

Table S3. Statistical significance of different factors on the short-term preference tests of Scnn1$^{++/++}$ and Scnn1$^{aa/bb}$ animals. After 4 weeks fed with sodium-adequate, low, or high salt diet, 10 to 11 Scnn1$^{++/++}$ and Scnn1$^{aa/bb}$ mice were subjected to short-term preference tests using an automated gustometer. To do so, animals were either restricted for 22.5 h with access to 2.0 mL water and 1 g food (attractive restriction conditions) or water-deprived for 22.5 h (aversive restriction conditions). Data represent the statistical significance (*p*-value) of diet, genotype, concentration, and a different combination of them, based on all tested concentrations of one substance (first 4 listed substances for each protocol were tested for 5 concentrations, whereas the remaining were only checked for 1 concentration). *p*-Values were based on UNIANOVA and post-hoc analysis using Bonferroni´s multiple comparison test. Differences were considered to be statistically significant if $p < 0.05$, as indicated in bold.

Protocol	Substance	Factor	*p*-Value
attractive	sucrose	diet	0.797
		concentration	**<0.001**
		diet X concentration	0.134
		genotype	0.743
		genotype X concentration	0.181
		diet X genotype	0.889
		diet X genotype X concentration	**0.034**
	MPG+IMP	diet	0.918
		concentration	**<0.001**
		diet X concentration	0.119
		genotype	**0.001**
		genotype X concentration	**0.005**
		diet X genotype	**0.031**
		diet X genotype X concentration	**0.026**
	NaCl	diet	0.360
		concentration	**0.030**
		diet X concentration	**0.005**
		genotype	**<0.001**
		genotype X concentration	0.640
		diet X genotype	**0.001**
		diet X genotype X concentration	**0.004**
	NaCl+amiloride	diet	0.075
		concentration	0.067
		diet X concentration	0.871
		genotype	**0.001**
		genotype X concentration	0.522

Protocol	Substance	Factor	*p*-Value
		diet X genotype	**<0.001**
		diet X genotype X concentration	0.974
	amiloride	diet	0.414
		genotype	**0.001**
		diet X genotype	**0.012**
	IMP	diet	0.380
		genotype	0.457
		diet X genotype	0.724
	denatonium	diet	0.293
		genotype	0.663
		diet X genotype	0.764
	citric aid	diet	0.113
		genotype	**0.006**
		diet X genotype	**0.009**
aversive	denatonium	diet	0.089
		concentration	**<0.001**
		diet X concentration	0.757
		genotype	**<0.001**
		genotype X concentration	**<0.001**
		diet X genotype	**<0.001**
		diet X genotype X concentration	**0.002**
	citric acid	diet	**0.015**
		concentration	**<0.001**
		diet X concentration	0.497
		genotype	**<0.001**
		genotype X concentration	**<0.001**
		diet X genotype	**<0.001**
		diet X genotype X concentration	**<0.001**
	NaCl	diet	**<0.001**
		concentration	**<0.001**
		diet X concentration	**0.012**
		genotype	0.060
		genotype X concentration	**0.004**
		diet X genotype	**<0.001**
		diet X genotype X concentration	**<0.001**
	NaCl+amiloride	diet	**<0.001**
		concentration	**<0.001**

**Protocol**	**Substance**	**Factor**	***p*-Value**
		diet X concentration	**<0.001**
		genotype	0.758
		genotype X concentration	0.132
		diet X genotype	**0.002**
		diet X genotype X concentration	**<0.001**
	amiloride	diet	0.389
		genotype	**0.002**
		diet X genotype	**0.028**
	sucrose	diet	**0.009**
		genotype	0.815
		diet X genotype	**0.007**
	sucralose	diet	0.080
		genotype	0.166
		diet X genotype	**0.025**
	MPG+IMP	diet	**0.001**
		genotype	**0.004**
		diet X genotype	**<0.001**

Table S4. Statistical significance of different factors on the short-term preference tests of Scnn1$^{++/++}$ and Scnn1$^{aa/bb}$ animals after amiloride intervention for 36 h. After receiving sodium-adequate diet, animals either had access to water or amiloride-containing water (300 µM), following short-term preference testing in an automated gustometer. Data represent the statistical significance (*p*-value) of an intervention (water with or without amiloride), genotype (Scnn1$^{++/++}$ versus Scnn1$^{aa/bb}$), and intervention X genotype on different taste solutions and their concentrations. Statistical testing was based on UNIANOVA and post-hoc analysis using Bonferroni´s multiple comparison test for data points of 10 to 16 animals. Differences were considered to be statistically significant if $p < 0.05$, as indicated in bold.

Substance	Concentration	Intervention	Genotype	Intervention X genotype
sucrose	10	**0.002**	0.160	**0.001**
	100	**0.007**	0.427	**0.002**
	1000	**<0.001**	0.776	**<0.001**
MPG+IMP	1	0.633	0.531	0.198
	10	**0.001**	**0.003**	**<0.001**
	100	**<0.001**	**0.036**	**<0.001**
NaCl	10	**0.008**	0.162	**0.033**
	100	**<0.001**	**0.001**	**<0.001**
	1000	**<0.001**	**0.011**	**<0.001**
NaCl+amiloride	10	**<0.001**	**0.019**	**<0.001**
	100	**<0.001**	0.525	**<0.001**
	1000	**0.002**	**0.030**	**0.002**
denatonium	10	0.583	0.599	0.684
citric aid	100	**0.006**	0.488	**0.026**

Table S5. Statistical significance of different factors on the short-term preference tests of $Scnn1^{++/++}$ and $Scnn1^{aa/bb}$ animals after access to amiloride-containing drinking water for 36 h. After receiving sodium-adequate diet, animals either had access to water or amiloride-containing water (300 µM), following short-term preference testing in an automated gustometer. Data represent the statistical significance (*p*-value) of intervention (water with or without amiloride), genotype, concentration, and different combination/interaction of them based on all 5 concentrations tested for 1 stimulus/substance (for denatonium and citric acid only 1 concentration was tested). Test was based on UNIANOVA and post-hoc analysis using Bonferroni´s multiple comparison test relying on data of 10 to 16 animals. Differences were considered to be statistically significant if $p < 0.05$, as indicated in bold.

Substance	Factor	*p*-Value
sucrose	intervention	**<0.001**
	concentration	**<0.001**
	intervention X concentration	**<0.001**
	genotype	0.998
	genotype X concentration	0.564
	intervention X genotype	**<0.001**
	intervention X genotype X concentration	**<0.001**
MPG+IMP	intervention	**<0.001**
	concentration	**<0.001**
	intervention X concentration	**<0.001**
	genotype	**0.001**
	genotype X concentration	0.119
	intervention X genotype	**<0.001**
	intervention X genotype X concentration	**0.001**
NaCl	intervention	**<0.001**
	concentration	**<0.001**
	intervention X concentration	**0.003**
	genotype	**<0.001**
	genotype X concentration	**0.019**
	intervention X genotype	**<0.001**
	intervention X genotype X concentration	**0.001**
NaCl+amiloride	intervention	**<0.001**
	concentration	**<0.001**
	intervention X concentration	**0.002**
	genotype	**0.007**
	genotype X concentration	0.364
	intervention X genotype	**<0.001**
	intervention X genotype X concentration	**0.014**
denatonium	intervention	0.583
	genotype	0.599
	intervention X genotype	0.684
citric aid	intervention	**0.006**
	genotype	0.488
	intervention X genotype	**0.026**
